# The anti-PD-1 era of cervical cancer: achievement, opportunity, and challenge

**DOI:** 10.3389/fimmu.2023.1195476

**Published:** 2023-07-25

**Authors:** Chen Li, Wei Cang, Yu Gu, Lihua Chen, Yang Xiang

**Affiliations:** ^1^ Department of Obstetrics and Gynecology, Peking Union Medical College Hospital, Chinese Academy of Medical Sciences and Peking Union Medical College, Beijing, China; ^2^ National Clinical Research Center for Obstetric & Gynecologic Diseases, Peking Union Medical College Hospital, Chinese Academy of Medical Sciences, Peking Union Medical College, Beijing, China

**Keywords:** anti-PD-1, cervical cancer, clinical trial, combination therapy, chemotherapy, immunotherapy, targeted therapy, radiotherapy

## Abstract

Cervical cancer is one of the three major female gynecological malignancies, becoming a major global health challenge. Although about 90% of early-stage patients can be cured by surgery, advanced-stage patients still need new treatment methods to improve their efficacy, especially for those with recurrence and metastasis tumors. Anti-PD-1 is currently the most widely used immune checkpoint inhibitor, which has revolutionized cancer therapy for different types of cancer. Pembrolizumab has been approved for second-line treatment of R/M CC but has a modest overall response rate of about 15%. Therefore, multiple types of anti-PD-1 have entered clinical trials successively and evaluated the efficacy in combination with chemotherapy, targeted therapy, and immunotherapy. At the same time, the dual specific antibody of PD-1/CTLA-4 was also used in clinical trials of cervical cancer, and the results showed better than anti-PD-1 monotherapy. In addition, anti-PD-1 has also been shown to sensitize radiotherapy. Therefore, understanding the current research progress of anti-PD-1 will better guide clinical application. This review summarizes ongoing clinical trials and published studies of anti-PD-1 monotherapy and combination therapy in the treatment of cervical cancer, as well as discusses the potential molecular biological mechanisms of combination, aiming to provide the basic evidence for support anti-PD-1 in the treatment of cervical cancer and new insights in combination immunotherapy.

## 1 Background

Cervical cancer (CC), one of the most common malignancies among females worldwide, ranks fourth for both incidence and mortality (1). For CC patients, different stages have different treatments and different outcomes. Early-stage CC (ESCC) (FIGO 2018 stage IA-IB2, IIA1) is usually treated with surgery with 5-year survival > 90% (2). Locally advanced CC (LACC) (FIGO 2018 stage IB3, IIA2-IVA) is typically treated with platinum-based concurrent chemoradiotherapy (CCRT), with 5-year survival 50 - 80% (3–5). However, survival decreases markedly for those who relapsed after previous standard treatment and patients diagnosed with metastatic disease (i.e., FIGO stage IVB), with a 5-year survival of only 10% (6, 7).

In recent years, the standard treatment for recurrent or metastatic cervical cancer (R/M CC) has been constantly updated, from platinum-based chemotherapy alone to chemotherapy +/- bevacizumab (Bev) based on the GOG-240 trial, and then to chemotherapy + pembrolizumab +/- Bev based on KEYNOTE-028/158/826 trials (6, 8–11). Meanwhile, cadonilimab, a PD-1-based bispecific antibody, was also approved for R/M CC in China in 2022 (12). In addition, there are many types of anti-PD-1 (such as nivolumab, balstilimab, cemiplimab, etc.) that entered clinical trials of cervical cancer in monotherapy or in combination with other regimes (e.g., targeted therapy, immunotherapy, chemotherapy, and radiotherapy et al.), and the clinical trial results of these anti-PD-1 have published results successively (9–11, 13–15).

Therefore, this review summarizes the latest clinical research progress of anti-PD-1 monotherapy or combination way in the treatment of R/M CC and LACC to better use anti-PD-1 for cervical cancer patients with different stages. Moreover, related mechanisms of anti-PD-1 combined with other therapies are given by preclinical studies in cancer therapy, hoping to provide biomarker information to guide the future use of anti-PD-1 and new molecular targets for combination therapy.

## 2 Immune checkpoint PD-1 and its antibodies treated for cervical cancer

Programmed cell death 1 (PD-1) was encoded by the *PDCD1/CD279* gene that was originally cloned by Honjo et al. (16). PD-1 could be induced on T and B lymphocytes by antigen receptor stimulation, and involved in the maintenance of immune tolerance serving as a negative regulator in T cell activation through the T cell receptor (TCR) and CD28 by engaging its ligands PD-L1/PD-L2 (17–19). With the exploration of the role of the PD-1 pathway in cancer, the evidence demonstrated that PD-1 blockade can reduce tumor burden by improving effector T cell function, alleviating T cell exhaustion, and reversing the dysfunctional state of tumor immune microenvironment (20, 21). Therefore, anti-PD-1 has become one of the most popular immunotherapy in the treatment of tumors in recent years (22).

Currently, there are about 18 types of anti-PD-1 registered in clinical trials to treat cervical cancer, and about 9 types of anti-PD-1 have published clinical trial results (**Table S1**).

## 3 Monotherapy of anti-PD-1 usually as the second-line therapy for R/M CC

Recurrent or metastatic cervical cancer (R/M CC) patients were generally referred to as those who have recurred and metastasized after radiation therapy in locally advanced patients, or who have been newly diagnosed with FIGO stage IV (6). If the first-line standard chemotherapy fails to control disease progression, anti-PD-1 becomes a remedy for patients with R/M CC.

In 2017, the results of a clinical trial of pembrolizumab as second-line therapy for R/M CC were published, opening a new chapter in the treatment of cervical cancer with anti-PD-1 (9). At present, there are five types of anti-PD-1 drugs have been used as second-line treatment for R/M CC patients with available clinical trial data, including pembrolizumab (9, 10), nivolumab (13, 23, 24), balstilimab (25), cemiplimab (26), and cadonilimab (12) (**Table 1**).

**Table 1:** Clinical trials data of anti-PD-1 monotherapy in the treatment of R/M CC.

Drug	Clinical trial identifier/No.	Phase	ORR					Median follow-up (months)	mDOR (months)	mPFS (months)	mOS (months)	Any grade TRAEs	Ref.
			Total	PD-L1^+^	PD-L1^-^	SCC	AC						
Pembrolizumab 10 mg/kg q2w	KEYNOTE-028 (NCT02054806)	Ib	17% (4/24)	17% (4/24)	–	–	–	11	5.4	2	11	75% (18/24)	(9)
Pembrolizumab 200 mg/kg q3w	KEYNOTE-158 (NCT02628067)	II	12.2% (12/98)	14.6% (12/82)	0% (0/15)	–	–	10.2	NR	2.1	9.4	65.3% (64/98)	(10)
Nivolumab 240 mg q2w	CheckMate-358 (NCT02488759)	I/II	26.3% (5/19)	20% (2/10)	16.7% (1/6)	–	–	19.2	NR	5.1	21.9	63.2% (12/19)	(13)
	JapicCTI-163212	II	25% (5/20)	33% (5/15)	0% (0/5)	–	–	8.6	–	5.6	–	65% (13/20)	(23)
Nivolumab 3 mg/kg q2w	NRG-GY002 (NCT02257528)	II	4% (1/25)	5.8% (1/17)	–	–	–	32	3.8	3.5	14.5	84% (21/25)	(24)
Balstilimab 3 mg/kg q2w	C-700-01 (NCT03104699)	II	15% (21/140)	20% (17/85)	7.9% (3/38)	17.6% (15/85)	12.5% (6/48)	14.6	15.4	–	–	71.4% (115/161)	(25)
Cemiplimab 350 mg q3w	GOG-3016 (NCT03257267)	III	16.4% (50/304)	18.3% (15/82)	11.4% (5/44)	–	–	18.2	–	8.4	12	88.3% (265/300)	(26)
Cadonilimab 6 mg/kg q2w	AK104-210-AU (NCT04380805)	II	33.3% (33/100)	43.8% (28/64)	16.7% (3/18)	34% (33/97)	0% (0/3)	–	–	–	–	91.9% (102/111)	(12)

ORR, objective response rate; mDOR, median durable of response; mPFS, median progression-free survival; mOS, median overall survival; IrAEs, immune-related adverse events; NR, not reach; SCC, Squamous Cell Carcinoma; AC, Adenocarcinoma; PD-L1, programmed death-ligand 1.

### 3.1 Pembrolizumab

Pembrolizumab (Keytruda^®^, Merck & Co., Inc.) was the first anti-PD-1 to be introduced into the KEYNOTE-028 and KEYNOTE-158 trials for the treatment of cervical cancer (9, 10).

KEYNOTE-028 was the first clinical trial of pembrolizumab in cervical cancer, aiming to explore the safety and efficacy of pembrolizumab monotherapy as a second-line treatment in PD-L1^+^ R/M CC patients. In this small sample trial, 24 patients were administrated pembrolizumab 10 mg/kg I.V. q2w. The ORR was 17% (4/24). Any grade of treatment-related adverse events (TRAEs) was 75% (18/24), and the most common were rash, pyrexia, and fatigue. KEYNOTE-028 suggested that pembrolizumab was well tolerated and has durable antitumor activity in patients with PD-L1^+^ advanced cervical cancer (NCT02054806) (9).

KEYNOTE-158 was a bigger cohort trial than KEYNOTE-028 that involved 98 patients including both PD-L1^+^ and PD-L1^-^. This study not only further verified the efficacy and safety of pembrolizumab but also compared the efficacy on patients with PD-L1^+^ or PD-L1^-^ tumors. The patients received pembrolizumab 200 mg I.V. q3w. The total ORR was 12.2% (12/98). In the PD-L1^+^ cohort, the ORR was 14.6% (12/82). In the PD-L1^-^ cohort, nobody has responded. Any grade TRAEs occurred in 65.3% (64/98) of patients, and the most common were hypothyroidism, decreased appetite, and fatigue. KEYNOTE-158 further verified the clinical activity of pembrolizumab, especially in the PD-L1^+^ cohort (NCT02628067) (10).

Therefore, the FDA accelerated the approval of pembrolizumab as a second-line treatment for PD-L1^+^ R/M CC patients in 2018 based on the above results (9, 10).

### 3.2 Nivolumab

Nivolumab (Opdivo®, Bristol-Myers Squibb Co.) has three clinical trials of monotherapy in R/M CC that published results, including CheckMate-358, JapicCTI‐163212, and NRG-GY002 trials (13, 23, 24).

CheckMate-358 trial results were published in 2019, in which 19 patients received nivolumab 240 mg I.V. q2w. The total ORR was 26.3% (5/19). The ORR in PD-L1^+^ and PD-L1^-^ cohorts was 20% (2/10) and 16.7% (1/6), respectively. Any grade TRAEs were 63.2% (12/19) of patients, and the most common were diarrhea, fatigue, and pneumonitis (NCT02488759) (13). In this trial, both PD-L1^+^ and PD-L1^-^ patients responded to nivolumab, which shows a difference from pembrolizumab.

JapicCTI‐163212 was another phase II trial of the nivolumab, in which 20 patients were also administrated with nivolumab 240 mg I.V. q2w. However, patients with PD-L1^-^ tumors have no response to nivolumab. The total ORR was 25% (5/25) that consistent with the CheckMate-358 generally. Notable, the ORR of the PD-L1^+^ cohort was higher reached to 33% (5/15) (23).

In addition, another trial NRG-GY002 showed disappointing outcomes compared to CheckMate-358 or JapicCTI‐163212, in which only one patient has reactivity to nivolumab 3 mg/kg I.V. q2w and a higher rate of TRAEs in 84% (NCT02257528) (24).

The difference between the above three trials may be due to the small sample size of patients involved. However, nivolumab can be used in PD-L1^-^ patients compared to pembrolizumab. In addition, nivolumab should be further introduced in larger sample clinical trials to verify its effectiveness.

### 3.3 Balstilimab

Balstilimab (AGEN2034, Agenus Inc.) showed superior rescue of antigen-specific T cell cytotoxicity *in vitro* relative to pembrolizumab, nivolumab reported by Joyce C. et al. (27).

C-700-01 trial was a larger phase II study published in 2021, in which patients received the recommended dose of balstilimab 3 mg/kg I.V. q2w based on the phase I study (28). The total OOR was 15% (21/140). In the PD-L1^+^ cohort, the ORR was 20% (17/85) which was higher compared to the PD-L1^-^ cohort (7.9%, 3/38). Any grade TRAEs was 71.4% (115/161), and the most common were asthenia, diarrhea, pyrexia, and fatigue. In addition, responses were observed across histologic subtypes, in which squamous cell carcinoma showed more sensitivity to anti-PD-1 compared to adenocarcinomas (ORR, 17.6%, 15/85 vs. 12.5%, 6/48) (NCT03104699) (25).

### 3.4 Cemiplimab

Cemiplimab (Libtayo®, Regeneron Pharmaceuticals Inc. & Sanofi Co.) showed improved survival than single-agent chemotherapy in the GOG-3016 trial.

GOG-3016 was a large, two-arms phase III study that published data in 2022. In this trial, a total of 608 patients were enrolled, who were randomly assigned to the group of cemiplimab 350 mg I.V. q3w or control therapy of single-agent chemotherapy (such as pemetrexed, topotecan, irinotecan, or gemcitabine, etc.) in a ratio of 1:1. In the overall population, the ORR was 16.4% (50/304) in the cemiplimab monotherapy group, as compared with 6.3% (19/304) in the single-agent chemotherapy group. Besides, the cohort of PD-L1^+^ patients showed more sensitivity with cemiplimab compared to PD-L1^-^ patients (ORR, 18.3%, 15/82 vs. 11.4%, 5/44).

For safety, any grade TRAEs was 88.3% (265/300) in the group of cemiplimab, which was safer than chemotherapy agent alone (91.4%, 265/290) (NCT03257267) (26).

### 3.5 Cadonilimab

Cadonilimab (AK104, Akeso, Inc.), a bispecific antibody targeting both PD-1 and CTLA-4 checkpoints, was designed to achieve preferential binding to tumor-infiltrating lymphocytes (TIL) rather than normal peripheral tissue lymphocytes (29).

AK104-201-AU study was the phase I b/II that showed good anti-tumor efficacy of cadonilimab 6 mg/kg q2w for up to 2 years, which was reported in the 2022 SGO meeting. The total ORR was 33% (33/100). The ORR in the PD-L1^+^ cohort was 43.8% (28/64) compared with 16.7% (3/18) in PD-L1^-^ cohort. Besides, the ORR in patients with squamous cell carcinoma was 34% (33/97) and 3 patients with adenocarcinoma had no response to cadonilimab. Any grade TRAEs was 91.9% (102/111); the most common were anemia, hypothyroidism, increased alanine aminotransferase (ALT), and increased aspartate aminotransferase (AST) (NCT04380805) (12).

Therefore, it was approved in China as the second/third-line treatment in R/M CC patients in June 2022, becoming the first bispecific antibody approved in cervical cancer (30).

## 4 Anti-PD-1 combined with other cancer therapeutic modalities for R/M CC

The efficacy of PD-1 monoclonal antibody monotherapy as second-line treatment in R/M CC patients was not very satisfactory indicated by the above research studies that ORR was only about 20% (9, 10, 25, 26). Hence, it is necessary to find a combination strategy to improve the efficacy of anti-PD-1. At present, anti-PD-1 in combination with chemotherapy, targeted therapy, and immunotherapy does increase the anti-tumor efficacy (**Table 2**).

**Table 2:** Clinical trials of anti-PD-1 combined with other therapy in the treatment of R/M CC.

Anti-PD-1	Combined therapy	Clinical trial identifier/No.	Phase	Arm	ORR									Any grade TRAEs		Ref.
					Total	PD-L1^+^	PD-L1^-^		SCC	AC		Ctrl		Anti-PD-1	Ctrl	
*Pembrolizumab 200 mg q3w	TP/TC +/- Bev	KEYNOTE-826 (NCT03635567)	III	Two	65.9% (203/308)	68.1% (186/273)	50.2% (138/275)		–	–		50.8% (157/309)		99.3% (305/307)	99.4% (307/309)	(11)
Serplulimab 4.5 mg/kg	albumin-bound paclitaxel	NCT04150575	II	single	52.4%	–	–		–	–		–		- -		(31)
Camrelizumab 200 mg q2w	apatinib	CLAP trial (NCT03816553)	II	single	55.6% (25/45)	69%	50%		77.8%	28.6%		–		95.6% (43/45)		(15)
Sintilimab 200 mg q3w	anlotinib	–	II	single	54.8% (23/42)	54.8% (23/42)	–		–	–		–		85.8% (36/42)		(32)
Balstilimab 3 mg/kg q2w	zalifrelimab	C-550-01 (NCT03495882)	I/II	single	25.6% (32/125)	32.8% (22/67)	9.1% (3/33)		32.6% (29/89)	8.8% (3/34)		–		71% (110/155)		(14)
Pembrolizumab 200 mg q3w	GX-188E	GX-188E-005 (NCT03444376)	II	single	42% (11/26)	–	–		–	–		–		44% (16/36)		(33)
Combined therapy	Combined therapy	Clinical trial identifier/No.	Phase	Arm	ORR (total)				ORR (PD-L1^+^)					Any grade TRAEs		Ref.
					A-15	A-10	B-10		A-15	A-10		B-10				
*Cadonilimab 10/15 mg/kg q2w	TP/TC +/- Bev	AK104-210 (NCT04868708)	II	three	73.3% (11/15)	68.8% (11/16)	92.3% (12/13)		70% (7/10)	75% (6/8)		88.9% (8/9)		95.6% (43/45)		(34)

ORR, objective response rate; mDOR, median durable of response; mPFS, median progression-free survival; mOS, median overall survival; TRAEs, treatment-related adverse events; NR, not reach; SCC, Squamous Cell Carcinoma; AC, Adenocarcinoma; PD-L1, programmed death-ligand 1; Ctrl, control group; TP/TC, paclitaxel + cisplatin/paclitaxel + carboplatin; Bev, bevacizumab; A-15, Cohort A-15; A-10, Cohort A-10; B-10, Cohort B-10.

* Represents that this combination therapy regimen was the first-line treatment for R/M CC patients.

### 4.1 Anti-PD-1 combined with chemotherapy for R/M CC

Systematic chemotherapy is a fundamental therapy for R/M CC, in which the first-line treatment options include cisplatin+ paclitaxel (TP) or carboplatin + paclitaxel (TC) +/- Bev, and the second-line treatment options include albumin-bound paclitaxel, topotecan, gemcitabine, etc. (35). In recent, anti-PD-1 combined with platinum-based chemotherapy +/- Bev was introduced as the first-line treatment for R/M CC, including pembrolizumab and cadonilimab (34, 36). In addition, anti-PD-1 combined with albumin-bound paclitaxel as second-line therapy for R/M CC also reported the results (31).

#### 4.1.1 Pembrolizumab plus platinum-based chemotherapy +/- Bev as first-line therapy

In 2021, the results of this phase III KEYNOTE-826 study were reported, in which 617 R/M CC patients have administrated pembrolizumab 200 mg q3w or placebo in a 1:1 ratio for up to 35 cycles combined with platinum-based chemotherapy, and +/- Bev per the investigator’s discretion. In the total cohort, the ORR in the group of pembrolizumab was 65.9% (203/308) compared to 50.8% (157/309) in the placebo. In the subset of pembrolizumab treatment, the ORR of the PD-L1^+^ cohort was significantly higher than that of PD-L1^-^ (68.1% vs. 50.2%). Any grade TRAEs in the pembrolizumab group were comparable with the control group (99.3% vs. 99.4%) (NCT03635567) (11) (**Table 2**).

**Figure 1:**
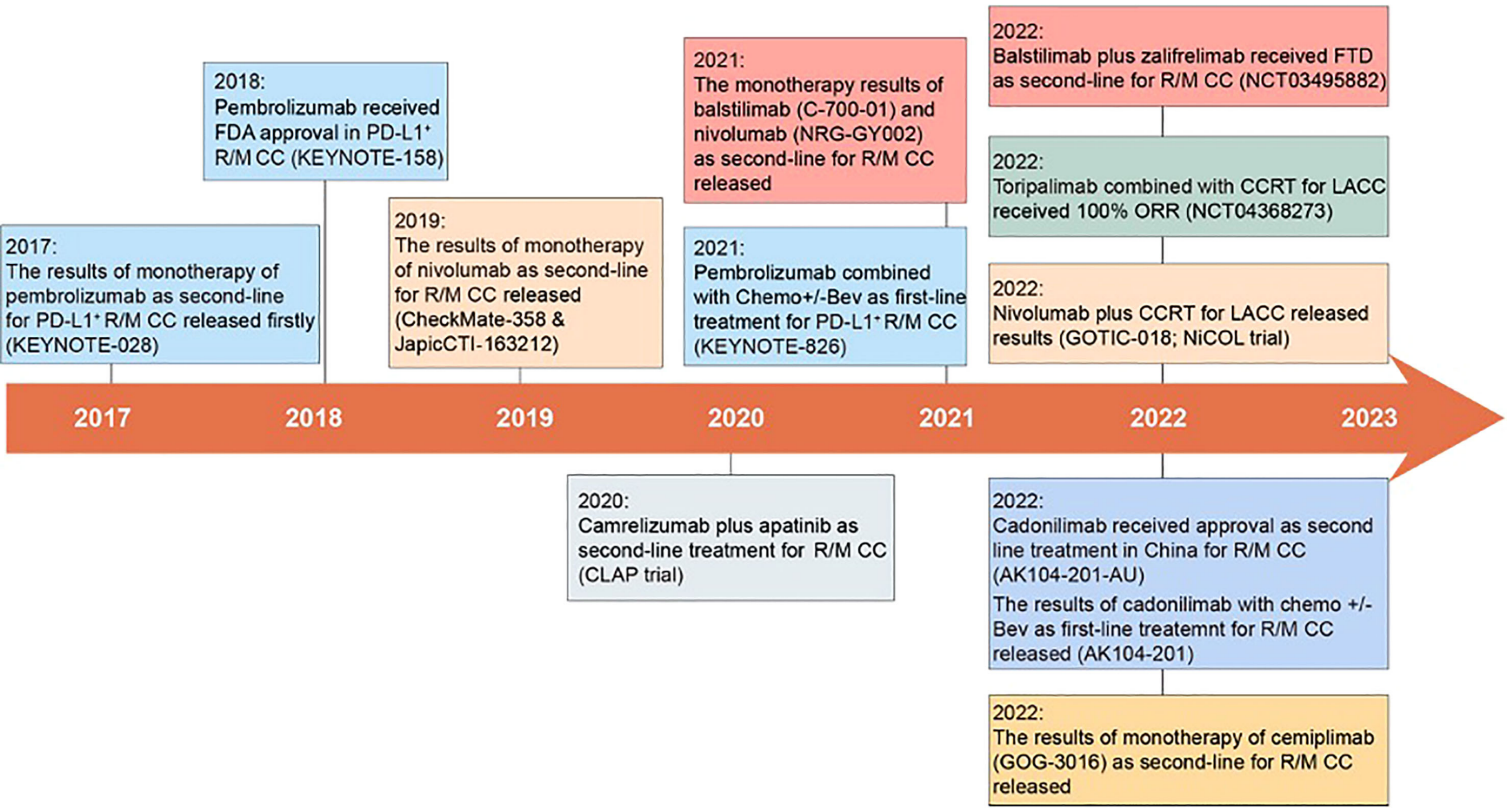
Milestones in the treatment of cervical cancer with anti-PD-1. In 2017, the first clinical trial of anti-PD-1 in R/M CC was published, opening a new chapter of anti-PD-1 in the treatment of cervical cancer (9). Subsequently, pembrolizumab monotherapy was approved by the FDA as a second-line treatment for R/M CC, and cadonilimab was approved in China (10, 30). Meanwhile, pembrolizumab in combination with platinum-based chemotherapy +/- Bev was approved as a first-line treatment for R/M CC (36). Many other anti-PD-1 (such as balstilimab, toripalimab, cemiplimab et al.) have also entered clinical trials and released results.

Based on KEYNOTE-826, the FDA approved pembrolizumab in combination with platinum-based chemotherapy +/- Bev as the first-line treatment for PD-L1^+^ (CPS ≥ 1%) R/M CC patients (36) (**Figure 1**).

#### 4.1.2 Cadonilimab plus platinum-based chemotherapy +/- Bev as first-line therapy

The phase II AK104-210 study results were reported at the ASCO annual meeting in 2022. The 45 enrolled patients were evenly assigned to 3 cohorts, 1) Cohort A-15: cadonilimab 15 mg/kg q3w + TP/TC; 2) Cohort A-10: cadonilimab 10 mg/kg q3w + TP/TC; 3) Cohort B-10: cadonilimab 10 mg/kg q3w + TP/TC + Bev 15 mg/kg, q3w.

The main results of ORR were 73.3% (11/15) in cohort A-15, 68.8% (11/16) in A-10, and 92.3% (12/13) in B-10, respectively, and the response to the treatment was regardless of CPS status of PD-L1. Any grade TRAEs occurred in 43 (95.6%) patients, which most commonly were anemia and leukopenia (34) (**Table 2**).

So far, cadonilimab has shown higher efficacy combined with platinum-based chemotherapy +/- Bev as a first-line treatment for R/M CC compared to pembrolizumab. However, the phase III long-term evaluation of cadonilimab needs an expanded sample and further follow-up (11, 34).

#### 4.1.3 Serplulimab plus albumin-bound paclitaxel as second-line therapy

This a two-stage, phase II study that planned to enroll 143 patients with PD-L1^+^ (CPS ≥1) who were given serplulimab (HLX10) 4.5 mg/kg plus albumin-bound paclitaxel 260 mg/m^2^ I.V. q3w. The stage I results (n = 20)were reported in the ASCO annual meeting in 2021, which showed a manageable safety profile that the most common grade ≥ 3 TRAEs were neutropenia, leukopenia, and anemia. No TEAEs led to drug discontinuation. The ORR was 52.4%. This trial represents a novel potential treatment option for R/M CC patients who have progressive disease or intolerable toxicity to first-line standard chemotherapy (NCT04150575) (31) (**Table 2**).

### 4.2 Anti-PD-1 combined with targeted therapy for R/M CC

At present, there are many types of targeted drugs combined with anti-PD-1 for R/M CC, including tyrosine kinase inhibitors (TKI), HADC inhibitors, PARP inhibitors, etc. However, only clinical trials of TKI combined with anti-PD-1 have published results so far, including camrelizumab combined with apatinib and sintilimab with anlotinib (**Table 2**).

#### 4.2.1 Anti-PD-1 combined with TKI (anti-VEGFR)

Tyrosine kinase inhibitors (TKI) are a class of compounds that can inhibit the activity of different receptor tyrosine kinases, such as EGFR, VEGFR, PDGFR, FGFR, and so on, in which targeting VEGFR was the main function used in cancer therapy (15, 32). In current, there are various TKI combined with anti-PD-1 under clinical study for cervical cancer treatment, including apatinib (VEFGR2/PDGFRα/c-kit), lucibanib (VEGFR1-3/FGFR1-2), anlotinib (VEGFR1-3/PDGFR/FGFR/c-Kit), et al.

##### 4.2.1.1 Camrelizumab plus apatinib as second-line therapy

The phase II CLAP trial showed that camrelizumab combined with apatinib had promising antitumor activity and manageable toxicities in R/M CC patients. The enrolled 45 patients were administrated camrelizumab 200 mg I.V. q2w plus apatinib 250 mg qd orally. The total ORR was 55.6% (25/45). In the PD-L1^+^ cohort, the ORR was 69% higher than 50% of PD-L1^-^. The 77.8% ORR was achieved for patients with SCC, and only 23% in patients with adenocarcinoma. TRAEs of ≥ 3 grade occurred in 32 patients (71.1%). Forty-three patients (95.6%) experienced at least one TRAEs, and the most common were hypertension, proteinuria, increased ALT, and increased AST (NCT03816553) (15) (**Table 2**).

##### 4.2.1.2 Sintilimab plus anlotinib as second-line therapy

The phase II study of sintilimab combined with anlotinib as second-line or later therapy has proven efficacious and safe for PD-L1^+^ R/M CC patients in 2022. The enrolled 42 patients were given sintilimab 200 mg on day 1 and anlotinib 10 mg qd on days 1-14 every 3 weeks. The ORR was 54.8% (23/42). Thirty-six patients (85.8%) experienced TRAEs, and the most common were hypothyroidism, increased AST, and hypertension (32) (**Table 2**).

As can be seen from the above studies, although camrelizumab + apatinib has achieved higher clinical activity, it has a higher proportion of side effects compared with sintilimab + anlotinib, which is mainly caused by apatinib (15, 32). Therefore, the dosage of antiangiogenic drugs should be closely monitored when combined to prevent side effects, rather than merely seeking efficacy.

Other trials of anti-PD-1 combined with different TKI in R/M CC were still undergoing, such as camrelizumab plus famitinib (NCT04906993), pembrolizumab plus lenvatinib (NCT04865887), sasanlimab plus axitinib (NCT04458259), tislelizumab plus sitravatinib (NCT05614453), etc. (**Table 3**).

**Table 3:** Current clinical trials of anti-PD-1 combined with other therapy in R/M CC.

Combined Therapies	Name	Drug	Anti-PD-1	Phase	Size	Clinical identifier	Ref.
Targeted therapy	TKI	Lenvatinib	Pembrolizumab	II	35	NCT04865887	–
		Axitinib	Sasanlimab	I	161	NCT04458259	–
		Apatinib	Camrelizumab	II	49	NCT03816553	(15)
		Famitinib		III	172	NCT04974944	–
		Anlotinib	Sintilimab	II	42	–	(32)
		Sitravatinib	Tislelizumab	II	57	NCT05614453	–
	HDACi	Chidamide	Toripalimab	I/II	40	NCT04651127	–
		Vorinostat	Pembrolizumab	II	112	NCT04357873	–
	PAPRi	Olaparib	Pembrolizumab	II	48	NCT04483544	–
	ADC	Tisotumab vedotin	Pembrolizumab	I/II	214	NCT03786081	–
Immunotherapy	Anti-CTLA-4	Zalifrelimab	Balstilimab	I/II	154	NCT03495882	(14)
	TIL	LN-145	Pembrolizumab	II	189	NCT03108495	–
	Vaccine	GX-188E	Pembrolizumab	II	60	NCT03444376	(33)
		ISA101b	Cemiplimab	II	105	NCT04646005	–
		HPV vaccine	Sintilimab	II	20	NCT04096911	–
Chemotherapy	Platinum-based chemotherapy +/- bevacizumab		*Pembrolizumab	III	617	NCT03635567	(11)
			*Serplulimab	II	48	NCT05444374	–
			*Prolgolimab	III	136	NCT03912415	–
			Prolgolimab	II	49	NCT03912402	–
			Tislelizumab	II	49	NCT05247619	–
	Platinum-based chemotherapy + bevacizumab		*Toripalimab	II	35	NCT04973904	–
			Pembrolizumab	II	40	NCT03367871	–
	Albumin-bound paclitaxel		Serplulimab	II	143	NCT04150575	(31)
			Camrelizumab	II	122	NCT05290935	–
	TC/TP		QL1604	II/III	458	NCT04864782	–

*Represents that this combination therapy regimen was the first-line treatment for R/M CC patients.

#### 4.2.2 Anti-PD-1 combined with HDACi

Histone deacetylase (HDAC) is an epigenetic regulatory enzyme that regulates the homeostasis of histone acetylation and histone deacetylation together with histone acetyltransferase (HAT), which is classified into four classes: class I was HDAC1-3/8, class II was HDAC4-7/9-10, class III enzymes was SIRTs, and class IV was HDAC11 (37, 38). Many HDAC inhibitors (e.g., trichostatin A, vorinostat, CG-745, and romidepsin) targeting different class HDAC have been shown to suppress cervical cancer cell proliferation by inducing cell-cycle arrest and apoptosis, stabilizing the expression of tumor suppressor p53 and Rb proteins and even inhibiting expression of HPV18 E6 and E7 genes (38–40).

Currently, the phase I/II clinical trials of HDACi combined with anti-PD-1 against R/M CC was recruiting and ongoing, including chidamide plus toripalimab (NCT04651127) and pembrolizumab plus vorinostat (NCT04357873) (**Table 3**).

#### 4.2.3 Anti-PD-1 combined with PARPi

Poly (ADP-ribose) polymerase inhibitor (PARPi) was primarily effective against BRCA-deficient ovarian or breast cancers by trapping PARP1 on damaged chromatin to achieve synthetic lethality (41). Recently, several studies explored the efficacy of PARPi in treating other cancers with no obvious BRCA mutations and demonstrated PARPi also modulates the tumor immune microenvironment (42, 43).

In the treatment of R/M CC, the combination strategy of olaparib plus pembrolizumab has entered a phase II clinical trial (NCT04483544) (**Table 3**).

Although clinical trial results of anti-PD-1 combined with HDACi or PARPi have not yet been published, this novel combination could be a new treatment option for R/M CC patients in the future, especially those who are intolerant to chemotherapy or antiangiogenic agents.

#### 4.2.4 Anti-PD-1 combined with antibody-drug conjugate

The antibody-drug conjugate (ADC) is composed of a monoclonal antibody, conjugated chain, and cytotoxic small molecule, which is a powerful anticancer drug. For example, tisotumab vedotin (HuMax^®^-TF-ADC, Tivdak™) is a tissue factor-directed antibody-drug conjugate that was engineered to target tumors expressing tissue factors, which has been approved by the FDA as the second-line therapy for R/M CC in 2021 (44).

At present, the phase I/II clinical trial of tisotumab vedotin combined with pembrolizumab was ongoing (NCT03786081) (**Table 3**).

### 4.3 Anti-PD-1 combined with immunotherapy for R/M CC

Dual immunotherapy is a new trend in cancer therapy, and the efficacy of anti-PD-1 combined with anti-CTLA-4 has been verified in a variety of tumors, such as melanoma (45), NSCLC (46) et al. In cervical cancer, balstilimab (anti-PD-1) plus zalifrelimab (anti-CTLA-4) has received fast-track designation (FTD) by the FDA as second-line therapy for R/M CC patients (47). In addition, anti-PD-1 combined with therapeutic HPV DNA vaccine has also shown advantages in the treatment of HPV-associated cervical cancer.

#### 4.3.1 Anti-PD-1 plus anti-CTLA-4 as second-line therapy

In 2022, the results of phase I/II C-550-01 study of balstilimab in combination with zalifrelimab in the treatment of R/M CC were reported (48). A total of 125 enrolled patients have administrated the combination dose of zalifrelimab 1 mg/kg I.V. q6w plus balstilimab 3 mg/kg I.V. q2w for up to 24 months. The total ORR was 25.6% (32/125). The ORR was 32.8% and 9.1% in patients with PD-1^+^ and PD-1^-^ tumors, respectively. The most common TRAEs of any grade were observed in 110 (71%) patients, including hypothyroidism, diarrhea, fatigue, and nausea (NCT03495882) (14) (**Table 2**).

#### 4.3.2 Anti-PD-1 plus HPV DNA vaccination as second-line therapy in HPV16/18^+^ R/M CC

In 2022, the phase II clinical study (GX-188E-005) results were released, which was conducted to explore the efficacy and safety of pembrolizumab plus GX-188E therapeutic DNA vaccine in R/M CC patients with HPV16/18 positive. A total of 36 patients enrolled, who received 2 mg GX-188E I.M. at 1, 2, 4, 7, 13, 19, and 46 weeks and pembrolizumab 200 mg q3w I.V. for up to 2 years or until disease progression. The interim analysis showed that the ORR was 42% (11/26). Any grade TRAEs was 44% (16/36), and no treatment-related deaths were reported. The interim data showed that pembrolizumab plus GX-188E therapeutic vaccine for HPV- positive R/M CC patients was safe and manageable. This trial project was still ongoing (GX-188E-005, NCT03444376) (33) (**Table 2**).

At present, other clinical trials of anti-PD-1 and vaccine combinations for cervical cancer include cemiplimab plus ISA101b vaccine (NCT04646005), and sintilimab plus HPV vaccine (NCT04096911) (**Table 3**).

#### 4.3.3 Anti-PD-1 plus tumor-infiltrating lymphocyte as second-line therapy

Tumor-infiltrating lymphocyte (TIL) therapy is a type of adoptive T cell therapy (ACT), which has proven efficacy in cervical cancer patients who were treated with a single infusion of HPV E6 and E7 reactivity TILs (HPV-TILs) and have experienced objective tumor responses with 33.3% ORR (3/9) (NCT01585428) (49). LN-145, designed by Iovance Co., has received FTD from the FDA as second-line therapy for R/M CC (50). A phase II trial of LN-145 combined with pembrolizumab in R/M CC with PD-L1 negative and MSI tumors were evaluated (NCT03108495) (**Table 3**).

## 5 Anti-PD-1 combined with concurrent chemoradiotherapy for LACC

Currently, clinical trials of CCRT combined with anti-PD-1 were being conducted in different modes of combination, including 1) Mode I: pre- and co-administration anti-PD-1 with CCRT followed by anti-PD-1 maintenance; 2) Mode II: co-administration anti-PD-1 with CCRT then anti-PD-1 maintenance; 3) Mode III: co-administration anti-PD-1 with CCRT simply, and the results are summarized below (**Table 4**).

**Table 4:** The combined modes of anti-PD-1 and CCRT in the treatment of LACC.

Three modes of anti-PD-1 plus CCRT	Anti-PD-1	Administration Dose	Maintenance dose and time	Phase	Sample	ORR	Clinical identifier	Ref.
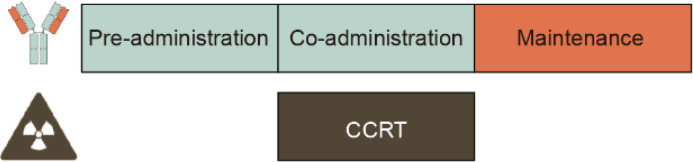	Nivolumab	240 mg/kg q2w	240 mg/kg q2w (52 weeks)	I	15 (cohort B)	–	GOTIC-018 NCT03298893	(51)
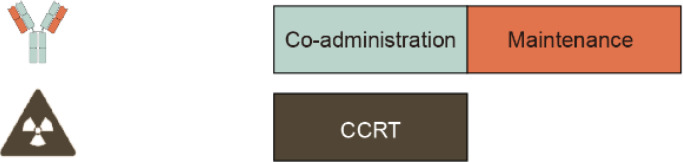	Nivolumab	240 mg/kg q2w	240 mg/kg q2w (52 weeks)	I	15 (cohort A)	–	GOTIC-018 NCT03298893	(51)
	Nivolumab	240 mg/kg q2w	240 mg/kg q2w (5 months)	I	16	93.8%	NiCOL NCT03298893	(52)
	Toripalimab	240 mg q3w	240 mg q6w (reach 1 year)	II	96	≈ 80%	NCT05084677	(53)
	Pembrolizumab	200 mg q3w	400 mg q6w (15 cycles)	III	980	–	KEYNOTE-A18, NCT04221945	–
	Camrelizumab	–	q2w until PD	II	92	–	NCT05311566	–
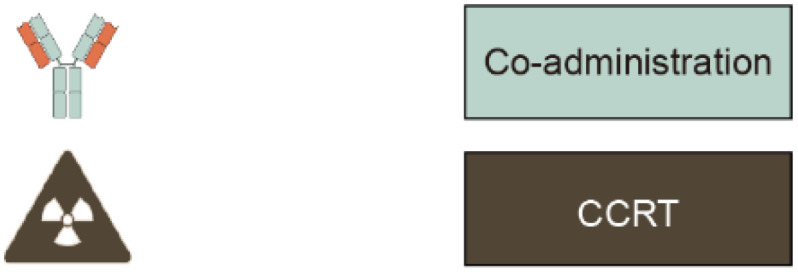	Toripalimab	240 mg days 1, 22, 43	–	I/II	22	100%	NCT04368273	(54)
	Cadonilimab	q3w	–	III	636	–	NCT05235516)	–

ORR, objective response rate; CCRT, concurrent chemoradiotherapy; PD, progressive disease.

### 5.1 Pre- and co-administration anti-PD-1 with CCRT followed by anti-PD-1 maintenance

#### 5.11 GOTIC-018 trial – cohort B

In 2022, Yabuno A. et al. reported interim data from a phase I trial evaluating the safety and feasibility of nivolumab combined with CCRT in LACC patients at the ASCO annual meeting (GOTIC-018; JMA-IIA00425; NCT03298893).

There are two processing models of treatment, in which cohort B enrolled 15 patients with pre-administration nivolumab before CCRT and then co-administration of nivolumab with CCRT followed by nivolumab maintenance. The CCRT regimen includes 4 or more cycles of cisplatin (40 mg/m2 q1w) and external beam radiotherapy (EBRT) followed by brachytherapy (BT). The nivolumab 240 mg/kg q2w maintenance therapy was scheduled for 52 weeks after completion of CCRT.

In cohort B, no patients required a break from EBRT and cisplatin dose reduction, and 1 patient required cisplatin discontinuation. The most common grade ≧ 3 AEs were neutropenia (26.7%), anemia (16.7%), and diarrhea (26.7%). Moreover, no patients required delay in starting CCRT due to the AEs related to the pre-administration of nivolumab, and no patients had PD before starting CCRT (51).

### 5.2 Co-administration anti-PD-1 with CCRT then anti-PD-1 maintenance

#### 5.2.1 GOTIC-018 trial – cohort A

In cohort A of GOTIC-018, another 15 patients with co-administration of nivolumab 240 mg/kg q2w with CCRT followed by maintenance therapy with nivolumab. The data showed that 2 patients required a break from EBRT, 2 required cisplatin dose reduction, and 1 required cisplatin discontinuation. The most common grade ≧ 3 AEs were neutropenia (60.0%), anemia (13.3%), and diarrhea (13.3%) (51).

Comparing the outcomes of the two cohorts in the GOTIC-018 trial, although the clinical activity was still unknown, the safety of cohort B showed better than that of cohort A (51).

#### 5.2.2 NiCOL trial: nivolumab combined with CCRT

The NiCOL trial of nivolumab combined with CCRT was also mode II. In this trial, the treatment way was the same as the GOTIC-018 trial, except nivolumab started on day 1 followed by 5 months.

A total of 16 patients were recruited, of which 3 patients experienced dose-limiting toxicity of cisplatin. In the period of receiving nivolumab maintenance, 1 patient developed a grade ≥ 3 diarrhea irAE. The ORR was 93.8% after BT completion. With a median follow-up of 16.6 months, and 1-year PFS was 81.2%. This study demonstrated that concurrent and maintenance nivolumab with CCRT appears to be a safe and feasible therapeutic option for LACC, associated with encouraging 1-year PFS rates (NiCOL trial, NCT03298893) (52).

#### 5.2.3 Toripalimab combined with CCRT

This phase II study explored the efficacy and tolerability of combining toripalimab with CCRT in the treatment of LACC patients. During CCRT, the enrolled patients received toripalimab 240 mg I.V. q3w. Maintenance treatment period, the patients were treated with toripalimab 240 mg q6w continuously until the whole treatment cycle reached 1 year. The exact results of this study have not yet been published by the researchers, but they assumed the primary endpoint ORR would yield 80% (NCT05084677) (53).

Other ongoing clinical trials of CCRT combined with anti-PD-1 according to mode II include KEYNOTE-A18, which is a large sample trial with 980 enrolled patients and pembrolizumab 200 mg I.V. q3w on day 1 for 5 cycles concurrent with CCRT followed by 400 mg I.V. q6w for an additional 15 cycles (KEYNOTE-A18, NCT04221945). Besides, a small sample trial of camrelizumab combined with CCRT was ongoing and camrelizumab was repeated q2w until disease progression (NCT05311566) (**Table 4**).

### 5.3 Co-administration anti-PD-1 with CCRT

A clinical trial conducted in mode III has already published the results in 2020 reported by Ou D et al, which was a phase I study of toripalimab combined with CCRT. The enrolled 22 patients received toripalimab 240 mg on days 1, 22, and 43 during CCRT.

The results showed that all patients have received CCRT successfully. The rate of grade ≥ 3 AEs was 45.5% (10/22), which most frequent was leukopenia (8/22, 36.4%). The most common irAE was hypothyroidism (2/22, 9.1%). The data showed that ORR was 100%. This study demonstrated a manageable safety profile and promising anti-tumor activity for LACC patients under the treatment of toripalimab combined with CCRT (NCT04368273) (54).

Other ongoing clinical trials according to mode III include AK104-305, a large sample trial with 636 enrolled patients who were administrated with cadonilimab I.V. q3w during CCRT (NCT05235516) (**Table 4**).

### 5.4 The timing of the combination of anti-PD-1 with CCRT was controversial

The above clinical trials with known results all have good reactivity and ORR of more than 80%, but no long-term follow-up evidence supports it. For safety, pre-administration with anti-PD-1 seems to have better security and does not cause dose-limiting toxicity. So, what is the better timing of anti-PD-1 combined with CCRT? The different preclinical studies present different timing suggestions for combination strategy in LACC (55–57).

One view was that anti-PD-1 prior to CCRT may benefit cervical cancer patients more. The dynamic alternation of tumor microenvironments (TEM) during CCRT was analyzed in 55 patients’ tumor samples. The results showed that CCRT will decrease the proportion of CD8^+^ effector T cells, increase Tregs, and reduce the expression of PD-1 in T cells and PD-L1 in tumor cells. Besides, TCR diversity also declined after CCRT, which was associated with worse PFS (55).

Another view suggested that combined with anti-PD-1 after or during CCRT treatment may have synergistic potential. The increasing soluble immune checkpoint protein (sICP) level (such as PD-1, PD-L1, LAG-3, TIM-3, CTLA-4, and so on) was detected in 51 patients’ serum before, during, and after CCRT. The results showed that CCRT might elevate these sICP levels in the patient’s serum. The increasing sICP level hints administration of immune checkpoint inhibitors after or during CCRT treatment may improve CCRT efficacy (56).

However, Chen J et al. has demonstrated that the timing of anti-PD-1 combined with CCRT was heterogeneous in different patients. They evaluated the immune markers of the patient’s tumor biopsies and analyzed the correlation with the short-term response. The data presented that tumor immune response to radiation before and after radiation had substantial heterogeneity, reflecting as CD8^+^ T-cell infiltration increased, decreased, and no significant change (57).

Therefore, the results of long-term follow-up from large-scale clinical trials were desirable. At the same time, it is recommended to dynamically monitor the changes in patients’ tumor immune microenvironments during treatment in order to excavate for molecular markers that may predict the therapeutic effect of anti-PD-1 combined with CCRT.

## 6 The reference mechanisms of anti-PD-1 combined with other therapies

**Figure 2:**
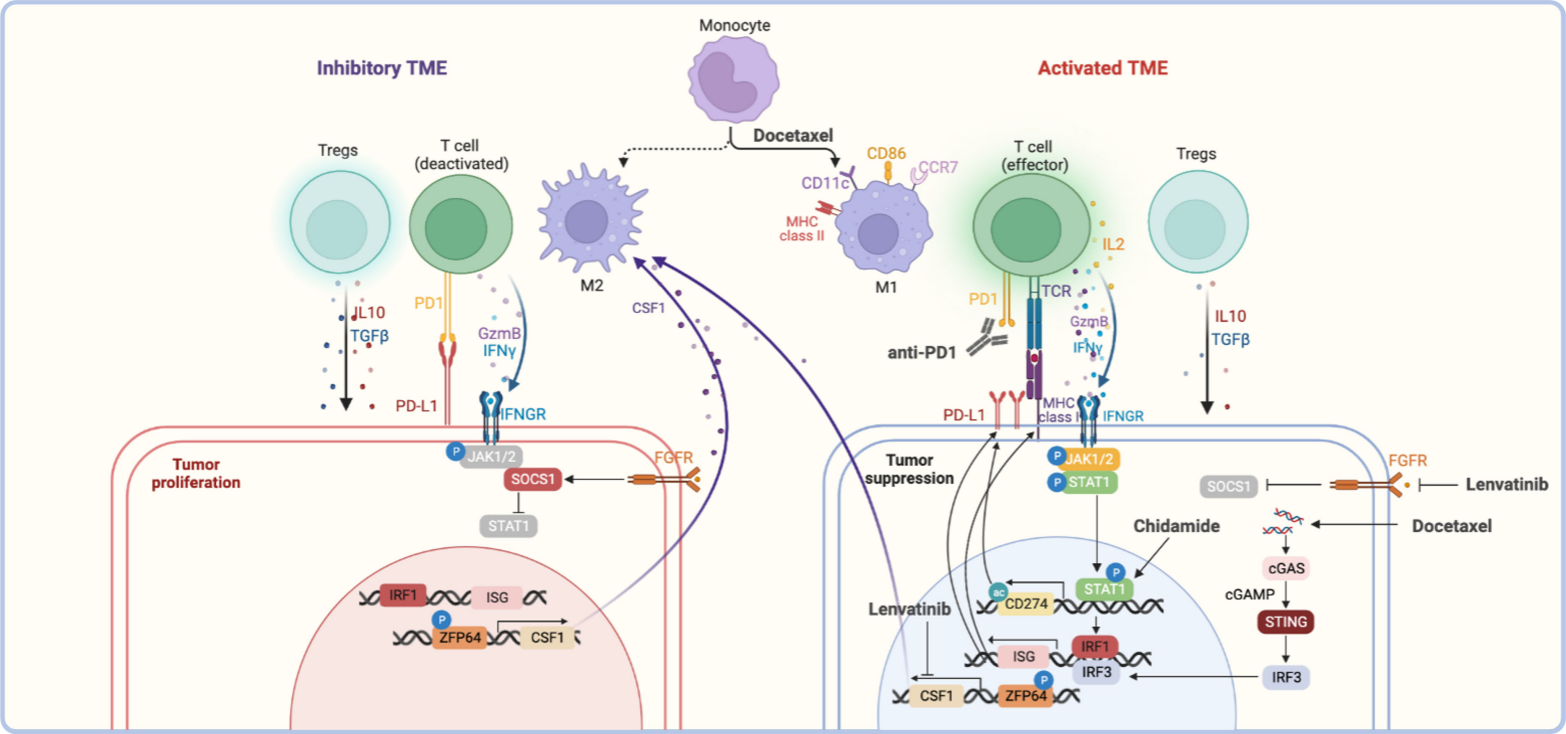
The reference mechanisms of anti-PD-1 combined with other therapeutic modalities. The anti-tumor effect of anti-PD-1 combined with other therapies was reflected in the activation of the tumor immune microenvironment, including activating effector T cells, polarizing M2 macrophages towards M1 type, and inhibiting Tregs function, etc., which together remodeling inhibitory TME (58–63). For other therapies, the upregulation of interferon-stimulated genes (ISGs) gene expression induced by the JAK/STAT signaling pathway or cGSA/STING signaling pathway was the basis of association, such as the upregulation of PD-L1 or MHC I was the common feature (58, 64–66). In addition, macrophage polarization was regulated by the ZFP64/CSF1 pathway, which balance of M1/M2 status was also the critical for anti-tumor effect (67).

Currently, pembrolizumab has been approved by the FDA for PD-L1^+^ (CPS ≥ 1) cervical cancer patients either as first-line or second-line treatment, which highlights the importance of PD-L1 expression in treatment with anti-PD-1 (10, 36). In addition, activated TME was fundamental to exert an anti-tumor effect. Therefore, the combination of anti-PD-1 with other therapies was mainly based on the activation of TME and the induction of PD-L1 expression to act as a complementary or synergistic effect (**Figure 2**).

### 6.1 The induction of PD-L1 expression

IFN-γ and TLR stimulation via a MEK/ERK-dependent and MyD88/TRAF6-dependent pathway were recognized as the key points of PD-L1 expression in plasma cells of multiple myeloma and melanoma (68, 69). PTEN loss also induces PD-L1 expression in glioma (70). At present, various treatments have been found to induce the expression of PD-L1 on the surface of tumor cells, which was the important reason for the combination with anti-PD-1.

The chemotherapeutic agent of paclitaxel and gemcitabine could induce both PD-L1 and MHC class I dependent on the NF-kB p65 signaling pathway in ovarian cancer cell lines, even in the absence of the IFN-γ signaling pathway (71). Besides, docetaxel also increased the expression of PD-L1 through ATM-NEMO- NF-κB signaling activity in prostate cancer (72).

Targeted therapy such as some TKI (e.g., apatinib, anlotinib, and lenvatinib) could promote the expression of PD-L1 through the IFNγ-stimulated JAK/STAT signaling pathway (58, 64, 65). PARPi could also induce PD-L1 expression via JAK2/STAT3 pathway in pancreatic cancer (73).. Besides, PARPi-mediated PD-L1 upregulation was associated with inactivated GSK3β that in turn upregulates PD-L1 (74). The HDACi could induce PD-L1 through epigenetic regulation way, for example, peak acetylation was observed at the first exon of the *CD274* in sarcoma cells after chidamide treatment (75).

### 6.2 The activated tumor immune microenvironment

The activated TME was the key to exerting an anti-tumor effect, which was reflected in the increasing proportion of activated effector T cells and M1-type macrophages, the decreasing of Tregs, MDSCs, and M2-types macrophages. The secretion of cytokines also changes, including increased levels of IFN-γ, TNF-α, and IL-2 as well as decreased levels of TGFβ and IL-6 (58–62). The studies have demonstrated that chemotherapies and targeted therapies could activate TME through different ways to synergist with anti-PD-1.

The cGAS-STING pathway activation could upregulate IFN-stimulated genes and promote T cell infiltration subsequently. The study showed that docetaxel may recruit T cells through the cGAS/STING-IFN pathway in prostate cancer, and combined with anti-PD-1 have improved the antitumor efficacy in a xenograft mouse model (66). In addition, docetaxel could polarize MDSCs toward an M1-like phenotype with upregulated markers such as MHC II, CD11c, and CD86 (63).

The study has shown that lenvatinib was critical for transforming macrophages to the M2 phenotype by targeting PKCα/ZFP64/CSF1 axis to trigger immune evasion and anti-PD-1 tolerance in a model of hepatocellular carcinoma (67). Moreover, the effect of angiogenesis inhibition of these TKI-VEGFR targeted drugs could also facilitate tumor vessel normalization by reducing CD31^+^ micro-vessels to regulate TME, which could be enhanced by anti-PD-1 further (61, 76).

The advantages of HDACi due to the increasingly global and enhancer acetylation of H3K27, which increased the expression of T-cell chemokines in multiple cell types (including cancer cells, macrophages, and T cells) to enhance T-cell infiltration and tumor regression (77). Besides, the upregulation of MHC I and MHC II genes after chidamide treatment indicated that HADCi might also enhance antigen presentation to promote the antitumor immune response (75).

## 7 Discussion

The clinical application trend of anti-PD-1 in cervical cancer is from monotherapy to combination therapy, from the way of second-line treatment to first-line treatment, and from treatment for those patients with R/M CC to LACC. Since 2017, anti-PD-1 has revolutionized the treatment of cervical cancer and renewed the NCCN guidelines now, the important milestone events of anti-PD-1 were summarized in this review (**Figure 1**). Then, the following four aspects of anti-PD-1 in the treatment of cervical cancer will be discussed, including clinical activity, safety, life quality, and cost-effectiveness.

The clinical activity of anti-PD-1 monotherapy as second-line treatment for R/M CC patients varied between different anti-PD-1 with ORR of 12.2% - 33.3%. Among them, the effect of dual-specific antibody cadonilimab was better with ORR of 33.3%; followed by nivolumab with ORR of about 25%; then pembrolizumab, balstilimab, and cemiplimab, the ORR were about 15% (9, 10, 12, 13, 23–26).

These ORR results of monotherapy were not satisfying. Thus, the combination way improved the efficacy of second-line anti-PD-1, such as in combination with TKI (e.g., camrelizumab plus apatinib, and sintilimab plus anlotinib) achieved the ORR of 54.8% - 55.6%; and in combination with chemotherapy (e.g., serplulimab plus albumin-bound paclitaxel) achieved the ORR of 52.4% (15, 31, 32). Besides, in combination with HPV DNA vaccination (pembrolizumab plus GX-188E) also improved ORR to 44% (33). However, in combination with anti-CTLA-4 (e.g., zalifrelimab plus balstilimab) only obtained ORR of 25.6%, which improved the clinical activity of zalifrelimab monotherapy, but the effect seems insufficient compared to PD-1/CTLA -4 dual specific antibody (14).

Upgrading from second-line treatment to first-line treatment is the trend of anti-PD-1. Anti-PD-1 is usually combined with standard chemotherapy +/- bevacizumab as a first-line agent. At present, the classic trial KEYNOTE-826 achieved ORR of 65.9% with longer PFS and OS compared to the control group (11). In recent, cadonilimab also entered the cervical cancer first-line trial, which reached ORR of 92.3% in a cohort of B-10 (34). This trial not only demonstrated the better clinical activity of cadonilimab but also the benefit of adding bevacizumab. In addition, the commonality of the results of these clinical trials showed that the ORR was higher in patients with PD-L1^+^ tumor than PD-L1^-^, and higher in patients with cervical squamous cell carcinoma than that of adenocarcinoma.

Therefore, once cervical cancer patients are diagnosed with recurrent or metastatic tumors, it is recommended to actively use PD-1 monoclonal antibody with reference to pathology and PD-L1 expression, preferably PD-1 dual-specific antibody, and in the case of tolerance, combined with bevacizumab may be a better choice. As for the dose of anti-PD-1, it is recommended to start at doses currently available in clinical trials and be adjusted depending on the patient’s tolerance at any time. Besides, the frequency of anti-PD-1 is also recommended to be once every two weeks or once every three weeks based on the patient’s condition.

The safety profile of anti-PD-1 was well established in all clinical trials currently. It is still necessary to know about the common TRAEs of anti-PD-1 for better management in clinical applications (**Table S1**). According to the present results, more than 60% of patients will experience any grade TAREs, in which combination therapy or a higher clinical efficacy is likely associated with a higher incidence of TRAEs.

In these trials, fatigue and asthenia are high-rate general disorders, so patients should be instructed to take adequate rest during drug administration. Gastrointestinal disorders occurred in almost all patients including decreased appetite, nausea, vomiting, diarrhea, constipation, and even enterocolitis, which require symptomatic management, including laxative or antidiarrheal treatment, and patients should eat a light diet. Hypothyroidism and hyperthyroidism are common endocrine disorders, so thyroid function tests should be paid more attention to every administration cycle or two-cycle. Anemia, leukopenia, and neutropenia are common TRAEs of the blood system, so a complete blood count should be tested closely. Hepatobiliary disorders including increased AST/ALT/γ-GGT/ALP and renal and urinary disorders including proteinuria and urinary tract infection, so liver and kidney function and urine routine tests should be tested regularly. Skin tissue disorders occurred in some patients including rash, dry skin, and pruritus, so patients should wear comfortable clothing and avoid exposure to strong sunlight. And if patients occurred in arthralgia and back pain, analgesic treatment can be considered. In combination with chemo-agents, the rate of alopecia, hypoesthesia, and peripheral neuropathy disorders may be increased inevitably. In combination with antiangiogenic agents may induce hypertension, so monitoring blood pressure is necessary in this way. Additionally, the judicious dose of antiangiogenic agents may be an important factor that needs to be carefully selected because excessive pruning vessels will result in hypoxia in the TME rendering the immune microenvironment in an inhibited state and weakening the anti-tumor ability (59). All in all, management and monitoring of TRAEs should be conducted throughout the treatment, which is not only based on the indicators of a series of laboratory tests but also on the observation of patients by the clinician.

The quality of life of female patients with cervical cancer who received anti-PD-1 or other systematic treatment was another aspect that needs to be concerned, especially in the deterioration symptoms of sexual dysfunction or sexual worry, menopausal symptoms, leg swelling, and peripheral neuropathy (78, 79). The latest published study reported the patients’ health-related quality of life with KEYNOTE-826. In the patient-reported outcomes (PROs) analyses, the median follow-up was 22 months, and 95% (587/617) of patients who received at least one dose of study treatment and completed at least one post-baseline PRO assessment were assessed. The data showed that compared to the placebo group, the median time to true deterioration (TTD) was longer in the pembrolizumab group. In addition, patients in the pembrolizumab group experience fewer cervical symptoms of lymphoedema, menopausal symptoms, and peripheral neuropathy than the placebo group (80). Therefore, anti-PD-1 treatment could improve OS and PFS and was not accompanied by a decrease in health-related quality of life in patients with recurrent, persistent, or metastatic cervical cancer. Besides, it is desirable to also assess the life quality with other anti-PD-1.

The cost-effectiveness of anti-PD-1 was also of great concern, though the safety, activity, and life quality were satisfactory in anti-PD-1 in cancer therapy. This year, two studies taken from the clinical data of the KEYNOTE-826 trial calculated the Quality-adjusted Life Years (QALYs) and evaluated the incremental cost-effectiveness ratio (ICER) of adding pembrolizumab to chemotherapy combinations in R/M CC patients from the Chinese and the United States (US) perspective, respectively. In the Chinese population, compared to the chemotherapy group, pembrolizumab plus chemotherapy contributed an incremental 1.12 QALYs with an incremental cost of $71,884.42, resulting in an ICER of $64,338.19, which was beyond the willingness-to-pay threshold of China (81). In the US, the data showed that the price of pembrolizumab had to reduce to at least $28.336 (55.8% of the current price) to make it 50% cost-effective (82). From these perspectives, the addition of pembrolizumab to chemotherapy is costly and might not be cost-effective for R/M CC.

However, another study showed that cemiplimab was a cost-effective treatment option for the second-line treatment of R/M CC patients as well as for patients with PD-L1 ≥ 1 tumor and all histological types from the American public payers’ perspective (83). Therefore, the cost-effectiveness analysis of other different anti-PD-1 in the treatment of cervical cancer is necessary, which could give clinicians a suggestion in terms of cost and better clinical application recommendations to patients with different economic incomes.

Of note, all clinical trials of anti-PD-1 in the first-second or second-line treatment of R/M CC required that the participants have not previously received any other immune checkpoint inhibitor. Therefore, it must be considered what treatment options are available to patients if anti-PD-1 therapy fails. How to solve the problem of anti-PD-1 resistance when used again is one of the directions that should be investigated in the future.

In combination anti-PD-1 with HADCi and PARPi are new combination ways for cervical cancer treatment. At present, the preclinical evidence has not been verified in the cervical cancer model, but we summarized the referential mechanism of combination in other cancer models. At the same time, the studies demonstrated that HADCi or PARPi were not only used in R/M CC combined with anti-PD-1 but also potentially sensitized radiotherapy for LACC patients (84–87). In addition, HDACi combined with anti-PD-1 may be a salvage treatment for patients who experienced anti-PD-1 resistance/refractory (88). Therefore, mechanistic studies should be validated before clinical application in the future.

Additionally, anti-PD-1 combined with CCRT for the treatment of LACC also obtained encouraged activity with ORR of more than 90% even reaching 100% ORR of toripalimab co-administered with CCRT (52, 54). However, the timing of administration was still controversial, so determining whether anti-PD-1 should be used for priming, intermittently, or sequentially in combination with CCRT needs to be precisely and comprehensively studied.

In conclusion, R/M CC patients will benefit from first-line therapy of anti-PD-1 combined with platinum-based chemotherapy definitely, and LACC patients who need CCRT treatment will also gain the potential benefit that can be verified further when the results of the KEYNOTE-A18 trial are published. In addition, though the activity and safety of some types of anti-PD-1 have been established, there is still insufficient long-term data available and most clinical trials of other different anti-PD-1 are still in phase I/II (**Table S2**).

Therefore, the following issues still need attention, 1) whether bevacizumab should be added to first-line therapy directly of anti-PD-1 combined with standard chemotherapy in the treatment of R/M CC needing further determined; 2) whether anti-PD-1 can be used again after the failure of the first-line anti-PD-1 therapy if changing the combination regimes; 3) whether maintenance therapy of anti-PD-1 should be continued after anti-PD-1 combined with CCRT. Moreover, exploring sensitivity markers that can predict the efficacy of anti-PD-1, either monotherapy or in combination way, should be investigated all along, which is one of the indispensable factors for the clinical use of anti-PD-1 normatively that can also improve cost-effectiveness inevitably. Paying attention to and regularly updating the clinical trials of anti-PD-1 in the treatment of cervical cancer will continue.

## Author contributions

CL and YX participated in the idea of the article; CL drafted the manuscript; WC, YG, and LC collected the related papers. CL and YX were responsible for the final review of the manuscript. All authors read and approved the final manuscript.
