# Supplementary material for: The anti-PD-1 era of cervical cancer: achievement, opportunity, and challenge
Source: Front Immunol. 2023 Jul 25;14:1195476. doi: 10.3389/fimmu.2023.1195476 (PMC10407549; doi:10.3389/fimmu.2023.1195476)
Supplement: Supplementary file 1 [file Table_1.docx]

**Table S1.** Any grade treatment-related adverse events of different anti-PD-1 in cervical cancer

| **Pembrolizumab** | | | |  |  |  | |  | |  | | |  | |  | | |  | |  |
| --- | --- | --- | --- | --- | --- | --- | --- | --- | --- | --- | --- | --- | --- | --- | --- | --- | --- | --- | --- | --- |
| **Trial** | **Way** | **N (%)** | **No.1** | **No.2** | **No.3** | **No.4** | | **No.5** | | **No.6** | | | **No.7** | | **No.8** | | | **No.9** | | **No.10** |
| KEYNOTE-028  (NCT02054806) | Alone | 75%  (18/24) | Rash  (5, 21%) | Pyrexia  (4, 17%) | Fatigue  (2, 8%) | Asthenia  (2, 8%) | | Constipation  (2, 8%) | | Diarrhea  (2, 8%) | | | Dry mouth  (2, 8%) | | Anemia  (2, 8%) | | | Proteinuria  (2, 8%) | | Dry skin  (2, 8%) |
| KEYNOTE-158 (NCT02628067) | Alone | 65.3%  (64/94) | Hypothyroidism  (10, 10.2%) | Decreased appetite  (9, 9.2%) | Fatigue  (9, 9.2%) | Diarrhea  (8,8.2%) | | Increased AST  (7, 7.1%) | | Asthenia  (7, 7.1%) | | | Pyrexia  (7, 7.1%) | | Hyperthyroidism  (7, 7.1%) | | | Arthralgia  (6, 6.1%) | | Nausea  (6, 6.1%) |
| KEYNOTE-826  (NCT03635567) | Plus  Chemotheapy+/-Bev | 99.3%  (305/307) | Anemia  (188, 61.2%) | Alopecia  (173, 56.4%) | Nausea  (122, 39.7%) | Diarrhea  (109, 35.5%) | | Fatigue  (88, 28.7%) | | Constipation  (87, 28.3%) | | | Arthralgia  (82, 26.7%) | | Peripheral neuropathy  (81, 26.4%) | | | Vomiting  (81, 26.4%) | Hypertension  (74, 24.1%) | |
| GX-188E-005  (NCT03444376) | Plus GX-188E | 44% (16/36) | Hypothyroidism  (4, 11%) | Diarrhea  (2, 6%) | Vomiting  (2, 6%) | Increased SCR  (2, 6%) | | Pruritus  (2, 6%) | | Constipation  (1, 3%) | | | Dry mouth  (1, 3%) | | Nausea  (1, 3%) | | | increased AST/ALT  (1, 3%) | | Rash  (1, 3%) |
| **Nivolumab** | | | |  |  |  | |  | |  | | |  | |  | | |  | |  |
| **Trial** | **Way** | **N (%)** | **No.1** | **No.2** | **No.3** | **No.4** | | **No.5** | | **No.6** | | | **No.7** | | **No.8** | | | **No.9** | | **No.10** |
| CheckMate-358  (NCT02488759) | Alone | 63.2%  (12/19) | Diarrhea  (4, 21.1%) | Fatigue  (3, 15.8%) | Pneumonitis  (2, 10.5%) | Abdominal pain  (2, 10.5%) | | Stomatitis  (2, 10.5%) | | Dry eye  (2, 10.5%) | | | Arthralgia  (2, 10.5%) | | Decreased  appetite  (1, 5.3%) | | | Hepatocellular injury  (1, 5.3%) | | - |
| JapicCTI-163212 | Alone | 65%  (13/20) | Increased AST  (3, 15%) | Hypothyroidism  (3, 15%) | Pruritus  (3, 15%) | Increased ALT  (2, 10%) | | Anemia  (2, 10%) | | Arthralgia  (2, 10%) | | | Diarrhea  (2, 10%) | | Pyrexia  (2, 10%) | | | Increased lipase  (2, 10%) | | Malaise  (2, 10%) |
| NRG-GY002  (NCT02257528) | Alone | 84%  (21/25) | Gastrointestinal disorders  (12, 48%) | Musculoskeletal and connective tissue disorders  (10, 40%) | Nervous system disorders  (9, 36%) | Respiratory, thoracic and mediastinal disorders  (9, 36%) | | General disorders and  administration  site conditions  (9, 36%) | | | Metabolism and nutrition disorders  (8, 32%) | | | Skin and subcutaneous tissue disorders  (7, 28%) | | | Vascular disorders  (7, 28%) | Infections and infestations  (5, 20%) | | Renal and urinary disorders  (4, 16%) |
| **Balstilimab** | | | |  |  |  | |  | |  | | |  | |  | | |  | |  |
| **Trial** | **Way** | **N (%)** | **No.1** | **No.2** | **No.3** | **No.4** | | **No.5** | | **No.6** | | | **No.7** | | **No.8** | | | **No.9** | | **No.10** |
| C-700-01  (NCT03104699) | Alone | 71.4%  (115/161) | Asthenia  (37, 23%) | Diarrhea  (20, 12.4%) | Pyrexia  (19, 11.8%) | Fatigue  (17,10.6%) | Hypothyroidism  (11, 6.8%) | | Hyperthyroidism  (6, 3.7%) | | | | | Pneumonitis  (5, 3.1%) | | | Enterocolitis  (5, 3.1%) | Arthralgia  (3, 1.9%) | | Pyrexia  (3, 1.9%) |
| C-550-01  (NCT03495882) | Plus  zalifrelimab | 71%  (110/155) | Hypothyroidism  (26, 16.8%) | Diarrhea  (22, 14.2%) | Fatigue  (18,11.6%) | Nausea  (14, 9%) | Hyperthyroidism  (6, 3.7%) | | | Increased AST  (13, 8.4%) | | | | Pyrexia  (13, 8.4%) | | | Anemia  (12, 7.7%) | Increased ALT  (11, 7.1%) | | Rash  (9, 5.8%) |
| **Cemiplimab** | | | |  |  |  | |  | |  | | |  | |  | | |  | |  |
| **Trial** | **Way** | **N (%)** | **No.1** | **No.2** | **No.3** | **No.4** | | **No.5** | | **No.6** | | | **No.7** | | **No.8** | | | **No.9** | | **No.10** |
| GOG-3016  (NCT03257267) | Alone | 88.3%  (265/300) | Anemia  (75, 25%) | Nausea  (55, 18.3%) | Fatigue  (50, 16.7%) | Vomiting  (48, 16%) | | Decreased appetite  (45, 15%) | | Constipation  (45, 15%) | | | Pyrexia  (35, 11.7%) | | Urinary tract infection  (35, 11.7%) | | | Asthenia  (33, 11%) | | Back pain (33, 11%) |
| **Cadonilimab** | | | |  |  |  | |  | |  | | |  | |  | | |  | |  |
| **Trial** | **Way** | **N (%)** | **No.1** | **No.2** | **No.3** | **No.4** | | **No.5** | | **No.6** | | | **No.7** | | **No.8** | | | **No.9** | | **No.10** |
| AK104-201-AU  (NCT04380805) | Alone | 91.9% (102/111) | Anemia  (37, 33.3%) | Hypothyroidism  (22, 19.8%) | Increased ALT  (20, 18%) | Increased AST  (18, 16.2%) | | leukopenia (16, 14.4%) | Hyperthyroidism  (16, 14.4%) | | | | | Pyrexia  (16,14.4%) | | Diarrhea  (13, 11.7%) | | Proteinuria  (12, 10.8%) | | Decreased appetite  (9, 8.1%) |
| AK104-210  (NCT04868708) | Plus  Chemotheapy+/-Bev | 95.6% (43/45) | Anemia  (55.6%) | Decreased blood cell count (46.7%) | Rash  (24.4%) | Hypoesthesia (24.4%) | | - | | - | | | - | | - | | | - | | - |
| **Camrelizumab** | | | |  |  |  | |  | |  | | |  | |  | | |  | |  |
| **Trial** | **Way** | **N (%)** | **No.1** | **No.2** | **No.3** | **No.4** | | **No.5** | | **No.6** | | | **No.7** | | **No.8** | | | **No.9** | | **No.10** |
| CLAP trial  (NCT03816553) | Plus  apatinib | 95.6% (43/45) | Hypertension  (19, 42.2%) | Proteinuria  (28.9%) | Increased AST  (16, 35.6%) | Increased ALT  (13, 28.9%) | | Anemia  (11, 24.4%) | | Fatigue  (11, 24.4%) | | | Rash  (7, 15.5%) | | Increased γ-GGT  (7, 15.6%) | | | Increased ALP  (7, 15.6%) | Neutropenia  (5, 11.1%) | |
| **Sintilimab** | | | |  |  |  | |  | |  | | |  | |  | | |  | |  |
| **Trial** | **Way** | **N (%)** | **No.1** | **No.2** | **No.3** | **No.4** | | **No.5** | | **No.6** | | | **No.7** | | **No.8** | | | **No.9** | | **No.10** |
| - | Plus  anlotinib | 85.8% (36/42) | Hypothyroidism  (14, 33.3%) | Increased AST  (9, 21.4%) | Hypertension  (8, 19%) | Diarrhea  (7,16.7%) | | Increased ALT  (7,16.7%) | | Hand-foot syndrome  (7,16.7%) | | Fistula  (6, 14.3%) | | | Hypertriglyceridemia  (6, 14.3%) | | | Anemia  (5, 11.9%) | Hypercholesterolemia  (5, 11.9%) | |
