# Supplementary material for: The anti-PD-1 era of cervical cancer: achievement, opportunity, and challenge
Source: Front Immunol. 2023 Jul 25;14:1195476. doi: 10.3389/fimmu.2023.1195476 (PMC10407549; doi:10.3389/fimmu.2023.1195476)
Supplement: Supplementary file 2 [file Table_2.docx]

**Table S2.** Ongoing clinical trials of anti-PD-1 monotherapy or combination therapy in cervical cancer

| **Toripalimab** | | |  |  |
| --- | --- | --- | --- | --- |
| **Phase** | **Trial ID** | **Status** | **Cancer type** | **Combined drug/therapy** |
| II | NCT04395612 | Unknow | R/M CC | Niraparib (PARPi) |
| I/II | NCT04651127 | Recruiting | R/M CC | Chidamide (HDACi) |
| II | NCT04973904 | Not yet recruiting | R/M CC | Paclitaxel+Cisplatin+Bevacizumab |
| I | NCT04731038 | Recruiting | R/M CC | Paclitaxel+Cisplatin/Carboplatin+Anlotinib |
| I | NCT05427396 | Recruiting | R/M CC | JS004 (Anti-BTLA) |
| II | NCT05342506 | Not yet recruiting | R/M CC | ScTIL (genetically modified TILs) |
| II | NCT05084677 | Recruiting | LACC | CCRT |
| I/II | NCT04368273 | Unknow | LACC | CCRT |
| **Sintilimab** | | |  |  |
| **Phase** | **Trial ID** | **Status** | **Cancer type** | **Combined drug/therapy** |
| II | NCT04590599 | Active, not recruiting | R/M CC | IBI310 (Anti-CTLA-4) |
| II | NCT04096911 | Unknow | R/M CC | quadrivalent HPV Vaccine |
| I/II | NCT05383482 | Recruiting | R/M CC | Afuresertib+ Nab-paclitaxel/Docetaxel |
| II | NCT04918628 | Recruiting | Stage IIIC2-IVB | CCRT |
| II | NCT05105672 | Recruiting | LACC | CCRT |
| **Camrelizumab (SHR-1210)** | | |  |  |
| **Phase** | **Trial ID** | **Status** | **Cancer type** | **Combined drug/therapy** |
| III | NCT04906993 | Enrolling by invitation | R/M CC | Famitinib malate |
| II | NCT04680988 | Active, not recruiting | R/M CC | Famitinib |
| II | NCT03827837 | Recruiting | R/M CC | Famitinib |
| II | NCT05234905 | Not yet recruiting | R/M CC | H101 (Oncolytic Virus) |
| II | NCT04974944 | Recruiting | R/M CC | Apatinib |
| II | NCT03816553 | Completed | R/M CC | Apatinib |
| I | NCT04508686 | Recruiting | R/M CC | Capecitabine |
| II | NCT04188860 | Completed | R/M CC | Abumin-bound paclitaxel |
| II | NCT05290935 | Recruiting | R/M CC | Albumin-bound paclitaxel |
| II | NCT04635956 | Unknow | R/M CC | Platinum/Etoposide/Bevacizumab |
| II | NCT05311566 | Recruiting | Stage IB2-IIIB | CCRT |
| II | NCT04974827 | Recruiting | LACC | CCRT |
| II | NCT04884906 | Recruiting | LACC | CCRT |
| II | NCT05151549 | Not yet recruiting | LACC | CCRT |
| II | NCT05554276 | Not yet recruiting | LACC | CCRT |
| **Balstilimab (AGEN2034)** | | |  |  |
| **Phase** | **Trial ID** | **Status** | **Cancer type** | **Combined drug/therapy** |
| I/II | NCT03104699 | Completed | R/M CC | - |
| III | NCT04943627 | Withdrawn | R/M CC | - |
| I/II | NCT03495882 | Completed | R/M CC | Zalifrelimab/AGEN1884 (Anti-CTLA-4) |
| II | NCT03894215 | Active, not recruiting | R/M CC | Zalifrelimab/AGEN1884 (Anti-CTLA-4) |
| II | NCT05033132 | Recruiting | R/M CC | Zalifrelimab/AGEN1884 (Anti-CTLA-4) |
| **Serplulimab ( HLX10)** | | |  |  |
| **Phase** | **Trial ID** | **Status** | **Cancer type** | **Combined drug/therapy** |
| II | NCT05444374 | Not yet recruiting | R/M CC | Paclitaxel+Cisplatin+Bevacizumab |
| II | NCT04150575 | Active, not recruiting | R/M CC | Albumin-bound paclitaxel |
| III | NCT04806945 | Withdrawn | R/M CC | Carboplatin/Cisplatin and Paclitaxel |
| III | NCT05173272 | Not yet recruiting | LACC | CCRT |
| **Zimberelimab (AB112)** | | |  |  |
| **Phase** | **Trial ID** | **Status** | **Cancer type** | **Combined drug/therapy** |
| I | NCT04772989 | Recruiting | R/M CC | AB308 (Anti-TIGIT) |
| II | NCT05824468 | Not yet recruiting | R/M CC | Lenvatinib |
| II | NCT05437692 | Recruiting | LACC | CCRT |
| **Candonilimab (AK104)** | | |  |  |
| **Phase** | **Trial ID** | **Status** | **Cancer type** | **Combined drug/therapy** |
| II | NCT05227651 | Not yet recruiting | CC | Radical surgery |
| II | NCT05817214 | Recruiting | R/M CC | Anlotinib |
| II | NCT05824494 | Not yet recruiting | R/M CC | Nab-paclitaxel |
| III | NCT04982237 | Recruiting | R/M CC | Paclitaxel+Cisplatin/Carboplatin±Bevacizumab |
| II | NCT04868708 | Active, not recruiting | R/M CC | Paclitaxel+Cisplatin/Carboplatin±Bevacizumab |
| II | NCT04380805 | Completed | R/M CC | - |
| II | NCT05063916 | Recruiting | R/M CC | - |
| III | NCT05235516 | Recruiting | LACC | CCRT |
| II | NCT05687851 | Recruiting | LACC | CCRT |
| **Tislelizumab (BGB-A317)** | | |  |  |
| **Phase** | **Trial ID** | **Status** | **Cancer type** | **Combined drug/therapy** |
| II | NCT05247619 | Recruiting | R/M CC | Paclitaxel+Cisplatin/Carboplatin+Bevacizumab |
| II | NCT05614453 | Not yet recruiting | R/M CC | Sitravatinib (TKI) |
| II | NCT04693234 | Active, not recruiting | R/M CC | Ociperlimab (Anti-TIGIT) |
| I | NCT05238883 | Recruiting | Advanced CC | HFB200301 (TNFR2 agonist antibody) |
| I | NCT05013268 | Not yet recruiting | LACC | Taxanes+Platinum |
| II | NCT05511623 | Not yet recruiting | Stage IIIC2 | CCRT |
| II | NCT05588219 | Recruiting | LACC | CCRT |
| II | NCT05310383 | Recruiting | R/M CC | Radiotherapy |
| **QL1604** | | |  |  |
| **Phase** | **Trial ID** | **Status** | **Cancer type** | **Combined drug/therapy** |
| II/III | NCT04864782 | Unknown | R/M CC | Paclitaxel+Cisplatin/Carboplatin |
| **SG001** | | |  |  |
| **Phase** | **Trial ID** | **Status** | **Cancer type** | **Combined drug/therapy** |
| II | NCT04886700 | Recruiting | R/M CC | - |
| I | NCT03852823 | Unknown | Advanced CC | - |
| III | NCT05715840 | Not yet recruiting | R/M CC | Paclitaxel+Cisplatin/Carboplatin±Bevacizumab |
| **Dostarlimab (TSR-042)** | | |  |  |
| **Phase** | **Trial ID** | **Status** | **Cancer type** | **Combined drug/therapy** |
| II | NCT04068753 | Recruiting | R/M CC | Niraparib |
| II | NCT05060432 | Recruiting | LACC | As maintenance therapy after CCRT |
| **Prolgolimab (BCD-100)** | | |  |  |
| **Phase** | **Trial ID** | **Status** | **Cancer type** | **Combined drug/therapy** |
| III | NCT03912415 | Recruiting | Advanced CC | Paclitaxel+Cisplatin/Carboplatin±Bevacizumab |
| II | NCT03912402 | Unknown | R/M CC | Paclitaxel+Cisplatin/Carboplatin+Bevacizumab |
| **Cemiplimab** | | |  |  |
| **Phase** | **Trial ID** | **Status** | **Cancer type** | **Combined drug/therapy** |
| III | NCT03257267 | Active, not recruiting | R/M CC | - |
| II | NCT04646005 | Not yet recruiting | R/M CC (HPV16+) | ISA101b vaccination |
| **Pembrolizumab (MK-3475)** | | |  |  |
| **Phase** | **Trial ID** | **Status** | **Cancer type** | **Combined drug/therapy** |
| III | NCT03635567 | Active, not recruiting | R/M CC | Paclitaxel+Cisplatin/Carboplatin±Bevacizumab |
| II | NCT03367871 | Terminated | R/M CC | Paclitaxel+Cisplatin/Carboplatin+Bevacizumab |
| II | NCT04641728 | Active, not recruiting | R/M CC | Olaparib (PARPi) |
| II | NCT04483544 | Recruiting | Advanced CC | Olaparib (PARPi) |
| II | NCT04865887 | Recruiting | R/M CC | Lenvatinib |
| II | NCT04230954 | Terminated | R/M CC | Cabozantinib (TKI) |
| I/II | NCT03786081 | Active, not recruiting | R/M CC | Tisotumab Vedotin |
| II | NCT03108495 | Recruiting | R/M CC | LN-145 (Autologous TILs) |
| I/II | NCT04652076 | Recruiting | R/M CC | NP137 (Anti-Netrin-1) |
| I | NCT05082259 | Recruiting | R/M CC | ASTX660 (Triple IAP blockade) |
| II | NCT04357873 | Active, not recruiting | R/M CC | Vorinostat (HDACi) |
| I/II | NCT05081609 | Recruiting | R/M CC | TransCon IL-2 β/γ |
| II | NCT02628067 | Recruiting | CC | - |
| I/II | NCT03476681 | Recruiting | CC | NEO-201 |
| II | NCT03192059 | Completed | CC | Radiation+Immune modulatory cocktail |
| I/II | NCT03917381 | Recruiting | CC | GEN1046 (Anti-PD-L1/4-1BB) |
| II | NCT03228667 | Active, not recruiting | CC | N-803 (IL-15 agonist antibody) |
| I | NCT05098132 | Recruiting | Advanced CC | STK-012 (IL-2 agonist antibody) |
| I | NCT04234113 | Recruiting | Advanced CC | SO-C101(IL-15 agonist antibody) |
| I | NCT03841110 | Completed | Advanced CC | FT500 (iPSC-derived NK cell) |
| I/II | NCT04301011 | Active, not recruiting | Advanced CC | TBio-651 (Oncolytic Vaccinia Virus) |
| I | NCT04432857 | Recruiting | Advanced CC | AN0025 (Anti-EP4) |
| I | NCT03849469 | Completed | Advanced CC | XmAb®22841(Anti-LAG3) |
| I | NCT05311618 | Recruiting | Advanced CC | NGM438 (Anti-LILRB4) |
| I | NCT05215574 | Recruiting | Advanced CC | NGM831 (Anti-ILT3) |
| I/II | NCT04913337 | Recruiting | Advanced CC | NGM707 (Anti-LILRB1/2) |
| I/II | NCT04140526 | Recruiting | Advanced CC | ONC-392 (Anti-CTLA-4) |
| I | NCT03454451 | Active, not recruiting | Advanced CC | CPI-006 (Anti-CD73) |
| I/II | NCT05187338 | Recruiting | Advanced CC | Ipilimumab+Durvalumab(Anti-PD-L1) |
| I/II | NCT03444376 | Active, not recruiting | Adcanced CC (HPV 16/18+) | GX-188E vaccination |
| II | NCT02635360 | Unknown | Advanced CC | CCRT |
| I | NCT03589339 | Recruiting | Advanced CC | NBTXR3 (nanoparticle)+Radiotherapy |
| II | NCT04238988 | Recruiting | LACC | Carboplatin+Paclitaxel → Radical surgery |
| III | NCT04221945 | Active, not recruiting | LACC | CCRT |
| **Nivolumab** | | |  |  |
| **Phase** | **Trial ID** | **Status** | **Cancer type** | **Combined drug/therapy** |
| II | NCT02257528 | Active, not recruiting | R/M CC | - |
| I | NCT03508570 | Active, not recruiting | R/M CC | Ipilimumab |
| I/II | NCT04042116 | Suspended | R/M CC | Lucitanib |
| I | NCT02379520 | Active, not recruiting | R/M CC (HPV+) | HPV-16/18 E6/E7-Specific T lymphocytes |
| I/II | NCT05180799 | Recruiting | Advanced CC | BA3071 (Anti-CTLA-4) |
| I | NCT03841110 | Completed | Advanced CC | FT500 (iPSC-derived NK cell) |
| I | NCT04925284 | Recruiting | Advanced CC | XB002 (TF-ADC drug) |
| I/II | NCT04895709 | Recruiting | Advanced CC | BMS-986340 (Anti-CCR8) |
| I | NCT03589339 | Recruiting | Advanced CC | NBTXR3(nanoparticle)+Radiotherapy |
| I/II | NCT03298893 | Completed | LACC | CCRT |
| II | NCT05492123 | Recruiting | LACC | Ipilimumab+CCRT |
| II | NCT03527264 | Terminated | LACC | CCRT |
| **Spartalizumab** | | |  |  |
| **Phase** | **Trial ID** | **Status** | **Cancer type** | **Combined drug/therapy** |
| II | NCT04802876 | Active, not recruiting | R/M CC | - |
| **Sym021** | | |  |  |
| **Phase** | **Trial ID** | **Status** | **Cancer type** | **Combined drug/therapy** |
| I | NCT04672434 | Recruiting | R/M CC | Sym024 (Anti-CD73) |
| **Sasanlimab** | | |  |  |
| **Phase** | **Trial ID** | **Status** | **Cancer type** | **Combined drug/therapy** |
| I | NCT04254107 | Recruiting | Advanced CC | SEA-TGT (Anti-TIGIT) |
| I | NCT04458259 | Recruiting | R/M CC | PF-07265807 (Anti-AXL/MER)±Axitinib |
